# The Rotational Spectrum of 2‑Furanyloxy Radical

**DOI:** 10.1021/acs.jpca.6c05049

**Published:** 2026-09-09

**Authors:** P. Bryan Changala, Michael C. McCarthy

**Affiliations:** † Center for Astrophysics, Harvard & Smithsonian, Cambridge, Massachusetts 02138, United States; ‡ 172322 JILA, University of Colorado Boulder and National Institute of Standards and Technology, Boulder, Colorado 80309, United States; § Department of Physics, University of Colorado Boulder, Boulder, Colorado 80309, United States

## Abstract

We report the hyperfine-resolved rotational spectrum of gas-phase 2-furanyloxy radical (^•^O–C_4_H_3_O) in the 6–36 GHz frequency range recorded using cavity Fourier transform microwave spectroscopy. These observations have yielded a precisely determined set of rotational constants, electron spin-rotation parameters, and proton hyperfine coupling constants. Our results enable a quantitative comparison of the structural and electronic properties of 2-furanyloxy with those of phenoxy and other closely related radicals, providing detailed physical insights into these prototypical resonance-stabilized π systems. The accurate laboratory rest frequencies derived from our measurements enable the quantification of the gas-phase chemistry of 2-furanyloxy in both the laboratory and in space, where the oxidation of aromatic rings remains poorly understood.

## Graphical abstract

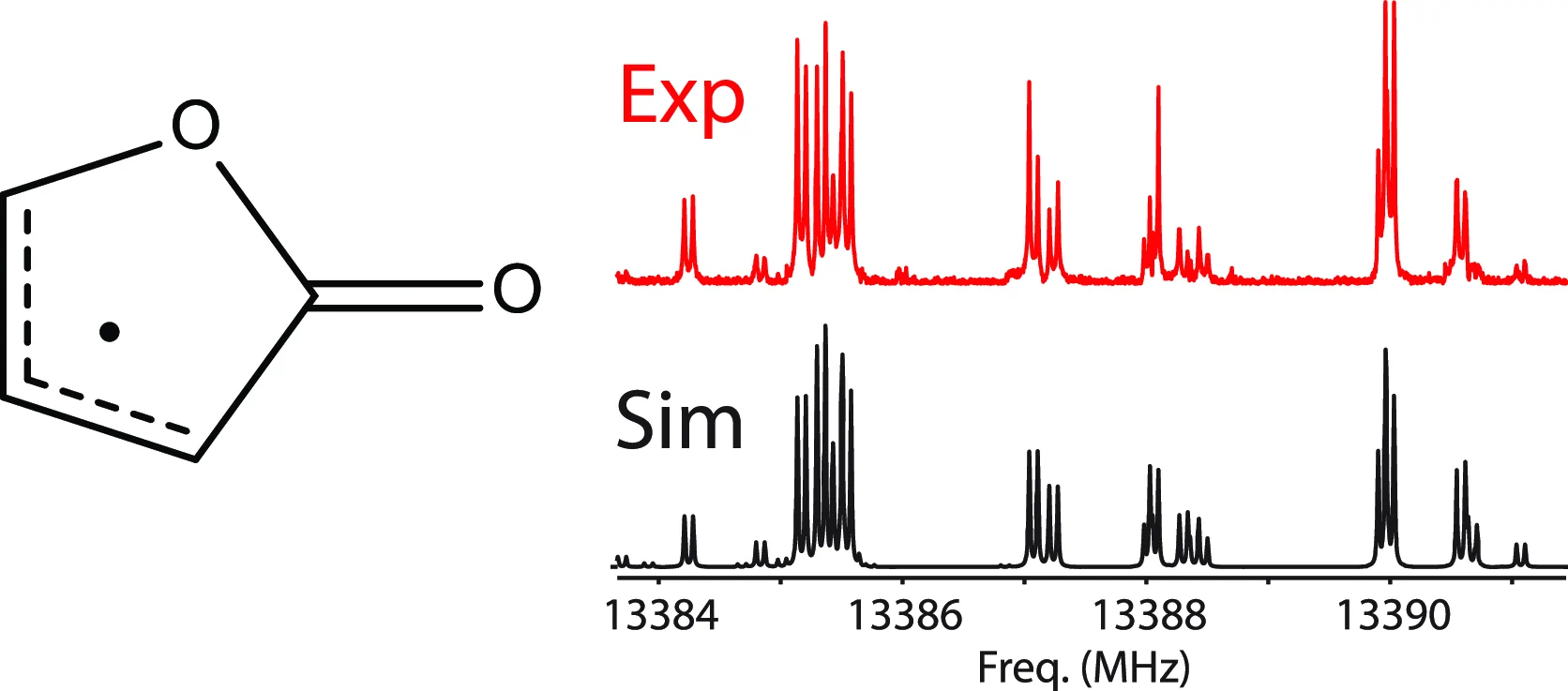

## Introduction

Oxygenated aromatic rings and heterocycles are a major component of coal, biomass, and other fuels. Insight into the thermal decomposition of these materials and the chemistry of their reactive intermediates can be obtained by detailed studies of small model compounds. 2-Methoxyfuran, the simplest furanic ether, is one such small molecule that has received particular interest. Gas-phase pyrolysis experiments observed 2-methoxyfuran to be exceptionally reactive compared to other five- and six-membered aromatic hydrocarbons and heterocycles.^1,2^ A quantum chemical study identified the unusually weak O–CH_3_ bond as the source of this reactivity and explained that the small bond dissociation energy (45 kcal/mol) is due to the resonance stabilization of the 2-furanyloxy radical (^•^O–C_4_H_3_O) produced by breaking this bond.^3^ Like other resonance-stabilized radicals, 2-furanyloxy is therefore expected to be a long-lived intermediate that can undergo further decomposition or secondary reactions.

Spectroscopy of 2-furanyloxy enables its quantification in reactive chemical environments and the characterization of its fundamental chemical and physical properties that influence its reactivity. There have, however, been few spectroscopic studies of this radical. Urness et al.^4^ measured the matrix infrared and photoionization spectra of 2-furanyloxy produced by the pyrolysis of 2-methoxyfuran. Zhang et al.^5^ have measured the photodetachment spectra of cryogenically cooled 2-furanyloxide anions, which allowed them to probe the vibrational structure of the ground and lowest excited electronic states of the neutral radical. The vibrational and electronic information obtained by these studies supported the findings of the earlier theoretical studies that 2-furanyloxy radical is stabilized by delocalization of the unpaired electron over the furanic ring and should be characterized as an allylic lactone.^3,6^ No other spectroscopic data on gas-phase 2-furanyloxy is known.

The characterization of the microwave spectrum of 2-furanyloxy would be particularly valuable. The rotational transition frequencies are closely related to its chemical structure, the precise measurement of which would provide evidence for its resonance-stabilized allylic lactone character. Moreover, small fine and hyperfine splittings in the rotational spectrum caused by interactions between the unpaired electron spin, proton spins, and the total rotational angular momentum are observable with the high spectroscopic resolution of cavity Fourier transform microwave (FTMW) spectrometers. The coupling parameters between these angular momenta are direct reporters of the open-shell electronic wavefunction and can be used to quantify the spatial distribution of the unpaired electron and coupling between the ground and excited electronic states of the radical. An accurately assigned and fitted hyperfine-resolved microwave spectrum of 2-furanyloxy would also provide the spectroscopic information needed to calculate its precise rest transition frequencies over the entire cm-wave region. This catalog would enable investigations of the chemistry and abundance of 2-furanyloxy in complex chemical environments both in the laboratory and in space by microwave spectroscopy. For example, although oxygen-bearing five- and six-membered rings have remained undetected in the gas phase in cold molecular clouds,^7^ it is known that oxygenated aromatic compounds form efficiently in astrochemical ices via oxygen-insertion mechanisms.^8^ Desorption of such ices in certain environments including protoplanetary disks might ultimately contribute to a detectable gas-phase inventory of oxygenated rings.^9^ The potential interstellar detection of 2-furanyloxy and related species by radio astronomy could therefore place important new constraints on the poorly understood oxidation chemistry of small aromatic compounds in astrophysical environments.

In this paper, we report the microwave rotational spectrum of 2-furanyloxy synthesized in a supersonic discharge expansion and measured by cavity FTMW spectroscopy in the 6 to 36 GHz frequency region. Its complex fine and hyperfine splitting patterns have been successfully assigned, and from these data we have derived accurate rotation, electron spin-rotation, and proton hyperfine coupling constants. Our experimental analysis is supported by theoretical structural and electronic properties calculated with highly correlated quantum chemical methods. These results provide detailed, quantitative insights into the structure of 2-furanyloxy, which we can now compare to other prototypical resonance-stabilized oxy radicals including phenoxy, the microwave spectrum of which has been recently measured by similar means.^10^

## Experimental Methods

The rotational spectrum of 2-furanyloxy was measured with a cavity FTMW spectrometer operating from 5 to 43 GHz.^11^ A dilute mixture of 2-methoxyfuran in neon (0.1%) at a total pressure of 2.5 kTorr was expanded from a solenoid valve in ca. 600 μs gas pulses at a rate of 5 Hz. The gas first flowed from the valve through two copper ring electrodes biased to a potential difference of 750 V, striking a 20–40 mA discharge, before supersonically expanding co-axially into a large vacuum microwave cavity and cooling to an internal rotational temperature of *T*_rot_ ≈ 2 K. 2-Furanyloxy was produced in the discharge by scission of the weak O–CH_3_ bond. This work together with our recent study of phenoxy produced via a discharge of anisole^10^ show that the highly non-thermal discharge plasma can mimic the thermal decomposition processes in pyrolysis reactor experiments, which have produced these radicals from the same alkoxy precursors.^4,12^ The discharge expansion source also produced a variety of oxygenated species that are known secondary products of the pyrolysis of 2-methoxyfuran, including crotonaldehyde and (*cis*-)acrolein, whose microwave spectra are already well characterized.^13,14^ We did not study the chemistry and abundance of these secondary products in our experiment in detail.

Rotational transitions of 2-furanyloxy were excited by a 1 μs pulse of resonant radiation, and the subsequent FID was detected by a sensitive microwave receiver and Fourier transformed. 2-Furanyloxy has a large permanent dipole moment aligned mostly parallel to the *a* principal inertial axis (μ_a_ ≈ 4.0 D; μ_b_ ≈ 1.0 D; ROHF-CCSD­(T)/cc-pCVTZ equilibrium values), and its strongest transitions were detected with a SNR of about 100 with 1 min of integration time over a 300 kHz instantaneous cavity bandwidth. Three mutually orthogonal pairs of Helmholtz coils surrounding the vacuum chamber were tuned to null the Earth’s magnetic field at the center of the microwave cavity. Zeeman shifts from the residual field at the edges of the cavity volume (<50 mG) lead to line broadening on the order of tens of kHz for the most field-sensitive transitions. The measured rest transition frequencies were derived from the mean of the manually determined maxima of the 2 symmetrically shifted Doppler components. This procedure has an accuracy of about 2 kHz.

## Theoretical Methods

The equilibrium geometry of 2-furanyloxy in its electronic ground state ( X̃A″2 ) was optimized using analytical gradients with coupled cluster theory including single, double, and perturbative triple excitations [CCSD­(T)].^15–18^ We employed both frozen-core (fc) and all-electron (ae) correlation treatments with the corresponding correlation consistent basis sets (cc-p­(C)­V*X*Z^19,20^ ) and restricted open-shell Hartree-Fock (ROHF) reference wavefunctions as implemented in the CFOUR program package.^21,22^ Zero-point vibrational corrections to the ground-state rotational constants were derived with second-order vibrational perturbation theory (VPT2)^23^ using cubic force constants calculated via finite differences. Electron spin-rotation tensor elements were computed using second-order property methods implemented for unrestricted Hartree-Fock (UHF) reference wavefunctions available in CFOUR.^24,25^ Proton hyperfine coupling parameters were also derived from the coupled-cluster unpaired spin density.

## Results

Our search for 2-furanyloxy was guided by the best-estimate theoretical predictions of the pure rotational frequencies derived from the quantum chemical estimates summarized in Table 1, which augment an initial fc-CCSD­(T)/cc-pVQZ optimized geometry with corrections for core–valence correlation, zero-point vibrational effects, and complete one-electron basis set extrapolations. We ultimately detected 19 rotational transitions (13 *a*-type, 6 *b*-type) from 6 to 36 GHz involving levels with asymmetric top quantum numbers *N* ≤ 5 and *K*_a_ ≤ 2. Each rotational transition appears as a dense cluster of lines split by fine and hyperfine interactions that couple the rotational angular momentum, the electron spin, and the 3 proton spins. Figure 1 shows an example of these splittings for the $N_{K_{a}K_{c}}=2_{02}-1_{01}$ rotational transition.

**Table 1:** Best-Estimate Theoretical Rotational Constants of 2-Furanyloxy^e^

parameter	reference^a^	core-correlation^b^	CBS^c^	zero-point^d^	total best-estimate
*A*	8925.0	+35.3	+12.1	–88.0	8884.3
*B*	4036.2	+15.5	+6.9	–19.9	4038.5
*C*	2779.3	+10.8	+4.4	–18.7	2775.7

a The equilibrium rotational constants at the fc-ROHF-CCSD­(T)/cc-pVQZ level of theory.

b The core–valence correlation correction calculated as the difference between the fc-ROHF-CCSD­(T)/cc-pVTZ and ae-ROHF-CCSD­(T)/cc-pCVTZ equilibrium rotational constants.

c The complete basis-set correction derived from a 3-point exponential extrapolation of the fc-ROHF-CCSD­(T)/cc-pV­(D,T,Q)­Z equilibrium rotational constants.

d The vibrational zero-point correction (e.g., *A*_0_ – *A*_e_) to the ground-state rotational constants derived with VPT2 using the fc-ROHF-CCSD­(T)/cc-pVTZ cubic force field.

e All values are in MHz.

**Figure 1:**
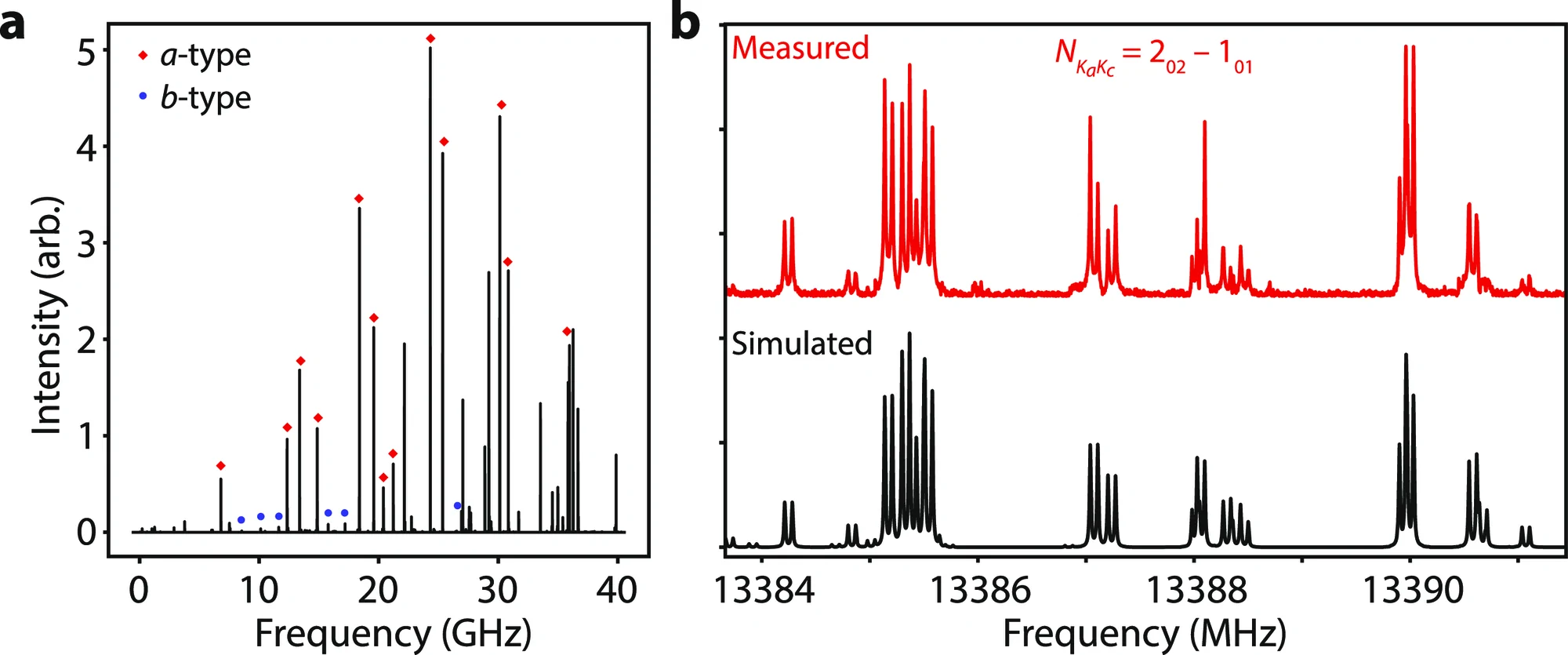
Microwave spectrum of 2-furanyloxy. (a) A simulated survey of the 0 to 40 GHz region with the measured *a*-type (red diamonds) and *b*-type (blue circles) transitions indicated. The simulation was performed at *T*_rot_ = 2 K. (b) The measured (upper, red) and simulated (lower, black) hyperfine structure of the $N_{K_{a}K_{c}}=2_{02}-1_{01}$ rotational transition near 13.4 GHz. Each hyperfine transition is symmetrically split into 2 Doppler components owing to the co-axial geometry of the jet expansion and microwave cavity axis. This 8 MHz-wide spectrum represents 27 concatenated 0.3 MHz cavity measurements, totaling 9 min of integration time.

The highly accurate coupled-cluster fine and hyperfine predictions made it straightforward to assign the individual hyperfine components of each rotational transition. We satisfactorily fitted 279 hyperfine transitions with an rms error of 1.6 kHz using a standard Watson-type Hamiltonian that included an asymmetric top semi-rigid rotor with quartic centrifugal distortion parameters (A-reduced, I^*r*^ representation) combined with 4 electron spin-rotation parameters (ϵ_aa_, ϵ_bb_, ϵ_cc_, ϵ_ab_) and 4 hyperfine coupling constants (*a*_F_, *T*_aa_, *T*_bb_ – *T*_cc_, *T*_ab_) for each proton. The off-diagonal fine and hyperfine coupling parameters involving the out-of-plane *c* axis (e.g., ϵ_ac_ and ϵ_bc_) are identically zero by planar symmetry. The best-fit parameters were optimized with the SPFIT program^26^ and are summarized in Table 2. The proton labeling scheme is defined in Figure 2. The SPFIT input and output files are available as Supporting Information.

**Table 2:** Spectroscopic Constants of 2-Furanyloxy in Its Ground Vibronic State Using an A-Reduced Hamiltonian in the I^*r*^ Representation^f^

parameter	measured^a^	theory
*A*	8879.52139(33)	8884.3^b^
*B*	4030.14567(11)	4038.5^b^
*C*	2771.420547(74)	2775.7^b^
Δ_*N*_ × 10^3^	0.3126(15)	0.3065^c^
Δ_*NK*_ × 10^3^	1.244(25)	1.216^c^
Δ_*K*_ × 10^3^	[2.150]	2.150^c^
δ_*N*_ × 10^3^	0.0922(12)	0.0906^c^
δ_*K*_ × 10^3^	[1.030]	1.030^c^
ϵ_aa_	–38.7335(13)	–35.673^d^
ϵ_bb_	–14.98401(66)	–13.914^d^
ϵ_cc_	–0.24542(41)	1.185^d^
(ϵ_ab_ + ϵ_ba_)/2	2.2042(96)	2.344^d^
*a*_F_(H_1_)	–37.5053(28)	–38.0838^e^
*T*_aa_(H_1_)	–3.2934(26)	–3.4174^e^
*T*_bb_ – *T*_cc_(H_1_)	1.3223(72)	1.9170^e^
*T*_ab_(H_1_)	–19.351(32)	–20.7283^e^
*a*_F_(H_2_)	2.6023(11)	3.6761^e^
*T*_aa_(H_2_)	0.2892(21)	–0.0675^e^
*T*_bb_ – *T*_cc_(H_2_)	6.6553(43)	7.0714^e^
*T*_ab_(H_2_)	1.890(22)	1.3759^e^
*a*_F_(H_3_)	–31.5745(29)	–32.1485^e^
*T*_aa_(H_3_)	–15.0897(25)	–16.5262^e^
*T*_bb_ – *T*_cc_(H_3_)	15.7462(63)	17.2094^e^
*T*_ab_(H_3_)	–7.100(29)	–7.7436^e^

a The 1σ uncertainty is shown in parentheses in units of the last digit. Values in square brackets were held fixed to the theoretical prediction.

b Best-estimate ground-state rotational constants (see Table 1).

c Harmonic centrifugal distortion parameters calculated at the fc-ROHF-CCSD­(T)/cc-pVTZ level of theory.

d Reduced electron spin-rotation tensor elements calculated at the fc-UHF-CCSD­(T)/cc-pVTZ level of theory. See text for a discussion of the reduction of this tensor.

e Hyperfine coupling constants calculated at the ae-ROHF-CCSD­(T)/cc-pCVTZ level of theory.

f All values are in MHz.

**Figure 2:**
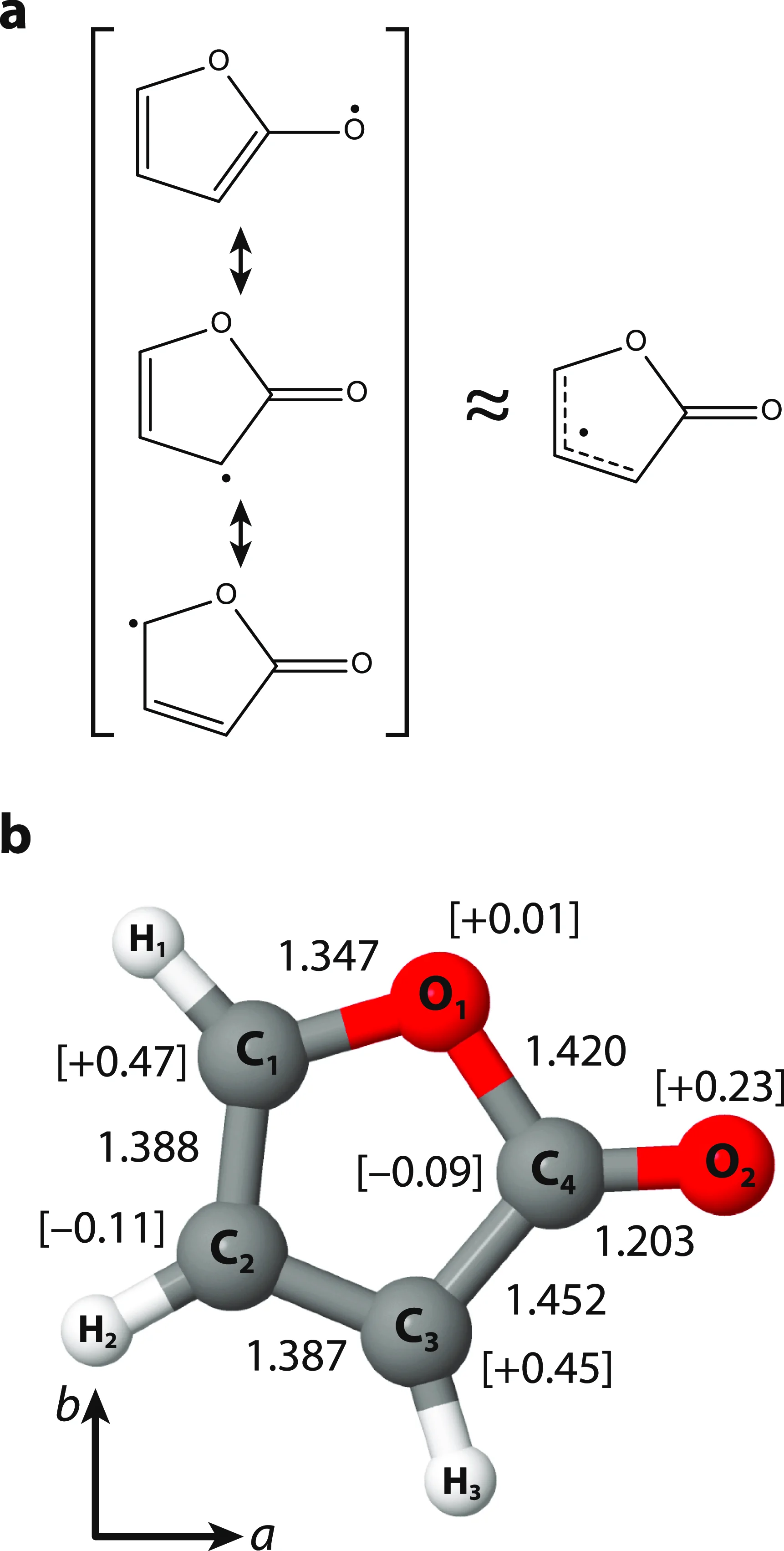
Structure and spin distribution of 2-furanyloxy. (a) The dominant resonance forms of 2-furanyloxy. (b) The calculated bond lengths of the heavy-atom skeleton are shown in Å (ae-ROHF-CCSD­(T)/cc-pCVTZ optimized structure). The Mulliken unpaired spin population for each heavy atom is shown in square brackets (derived from the ae-ROHF-CCSD­(T)/cc-pCVTZ correlated spin density). The proton indices in Table 2 correspond to the labels shown here. The orientation of the *a* and *b* inertial axes is also indicated.

Note that the theoretical spin-rotation tensor elements reported in Table 2 have been appropriately reduced in order to compare to the experimentally determinable values.^27^ We have made the standard choice of reduction that ϵ_ab_ = ϵ_ba_. The unreduced ab initio off-diagonal elements are ϵ_ab_ = 2.990 MHz and ϵ_ba_ = 2.052 MHz. The unreduced diagonal elements (ϵ_aa_, ϵ_bb_, and ϵ_cc_) differ from their reduced values by less than 10^–3^ MHz.

## Discussion

The resonance structures of 2-furanyloxy are illustrated in Figure 2a. These diagrams predict that the dominant bonding arrangement is that of an allylic lactone with the unpaired electron mostly located on the allylic fragment (C_1,2,3_), with a minor contribution from the oxy (O_2_) radical form. Our experimental results provide direct probes of the chemical structure and ground-state electronic properties that corroborate this picture.

The close agreement between the theoretical and measured rotational constants experimentally validate the calculated equilibrium structural parameters (Figure 2b), which are similar to those derived from DFT calculations reported previously.^3,5^ As expected from the π-delocalized electronic structure, the C_1_–C_2_ and C_2_–C_3_ bond lengths are calculated to be nearly equal (ca. 1.388 Å) and consistent with an approximate bond order of 1.5 between sp^2^-hybridized carbon atoms. The semi-experimental equilibrium single C–C and double C=C bond lengths in *trans*-1,3-butadiene,^28^ for example, are 1.454(1) Å and 1.338(1) Å, respectively, which average to 1.396 Å. It would be illuminating to compare these allylic bond lengths to those of allyl radical itself, but its experimental microwave structure is not yet known. An alternative experimental comparison is to the CH_2_–CCH bond length of propargyl radical, which also has a nominal bond order of 1.5 due to π-delocalization of the unpaired electron (i.e., ĊH_2_–C≡CH ↔ CH_2_=C=ĊH). The semi-experimental equilibrium value of this parameter is 1.3747(5) Å,^29^ only 1% shorter than the computed allylic bond lengths in 2-furanyloxy. In fact, these differences are already comparable to the expected theoretical errors of the ae-ROHF-CCSD­(T)/cc-pCVTZ bond lengths shown in Figure 2. (For example, the largest remaining correction is likely that from extrapolating to the complete one-electron basis set limit, which decreases the computed bond lengths by a further 3 mÅ.) The resonance diagrams also predict a near-double bond for C_4_=O_2_, consistent with its short bond length of 1.203 Å. (Compare that of H_2_C=O, 1.205 Å.^30^)

The measured proton hyperfine coupling constants provide additional physical insight into resonance-delocalized electronic structure. As is well known from electron spin resonance and microwave spectroscopy of π radicals, the isotropic hyperfine coupling constants (*a*_F_) of H atoms bonded to sp^2^ C atoms are approximately equal to −63 MHz × *ρ*_π_, where *ρ*_π_ is the unpaired spin population on the C atom.^31^ Estimating *ρ*_π_ with the Mulliken atomic spin populations derived from the CCSD­(T) unpaired spin density (Figure 2b), we would expect the *a*_F_ coupling constants of H_1_, H_2_, and H_3_ to be −30 MHz, +7 MHz, and −28 MHz, in reasonable qualitative agreement with both the measured and ab initio values (Table 2).

A similar pattern is observed in the anisotropic hyperfine coupling constants. Because all 4 independent matrix elements of the *T*_*ij*_ tensor have been measured for each proton, we can derive their principal eigenvalues. We focus on the positive (negative) in-plane principal value for the terminal (central) positions of the allylic unit, C_1_–C_2_–C_3_. In the model developed by McConnell and Strathdee (MS),^32^ this principal value corresponds to the anisotropic coupling parameter parallel to the local CH bond direction. The experimentally derived values are 19.1 MHz, −0.64 MHz, and 17.0 MHz for H_1_, H_2_, and H_3_, respectively. The MS model predicts that this principal value should be approximately 43 MHz × ρ_π_, i.e., 20.2 MHz, −4.73 MHz, and 19.4 MHz, respectively. As with the isotropic hyperfine coupling constants, these simple model relations are least reliable for the central position of the allylic unit. This failure is unsurprising: the models were derived for CH fragments with carbon π spin populations near ρ_π_ ≈ +1, but C_2_ has only a small, negative spin population (ca. −0.1). Both the adjacent C_2_ atom and next-nearest neighbor C_1,3_ atoms will therefore have comparable contributions to the H_2_ hyperfine coupling parameters.

Both the resonance structures and the ab initio electronic wavefunction predict a small but significant unpaired spin density on the oxy (O_2_) oxygen (ρ ≈ +0.23). Although we lack nuclear hyperfine probes of the unpaired electron density on this atom, we can still gain insight into the O_2_ spin population through the spin-rotation fine structure parameters (ϵ_*ij*_). The primary contributions to ϵ_*ij*_ are second-order interactions between the ground electronic state and excited electronic states connected by simultaneous spin–orbit and electronic orbital angular momentum matrix elements.^33^ O-centered contributions to these matrix elements tend to dominate because the atomic spin–orbit coupling parameter of O is about 5× larger than that of C. Following similar arguments we made in the analysis of phenoxy radical,^10^ a single-center, single-perturber approximation based on these physical trends (as well as the fact that the C_4_–O_2_ bond is nearly parallel with the inertial *a* axis) allows us to roughly estimate the ϵ_*ij*_ tensor elements as

$$\begin{array}{ll} \epsilon_{aa} & \approx 4\zeta_{O}A\frac{\rho_{X}\rho_{B_{2}}}{E_{B_{2}}-E_{X}},\\ \epsilon_{bb} & \approx 4\zeta_{O}B\frac{\rho_{X}\rho_{A_{1}}}{E_{A_{1}}-E_{X}},\\ \epsilon_{cc} & \approx \epsilon_{ab}\approx 0, \end{array}$$

where ζ_O_ ≈ −139 cm^–1^ is the effective O­(2p) atomic spin–orbit constant and *A* and *B* are the rotational constants. ρ_X_ and *E*_X_ refer to the O atomic spin population and energy of the ground electronic state X̃A″2 . “B_2_” refers to the excited electronic state that moves the electron hole to the in-plane 2p orbital of the O_2_ atom aligned mostly along the *b* axis (i.e., correlating to the 1^2^B_2_ state of phenoxy radical), and “A_1_” refers to the excited electronic state that places the electron hole in the in-plane 2p orbital aligned mostly along the *a* axis (i.e., correlating to the 1^2^A_1_ state of phenoxy radical). Photodetachment spectroscopy^5^ has identified the “B_2_” state as the first excited ÃA′2 state of 2-furanyloxy, which has a measured adiabatic electronic excitation energy of 2.14 eV. Taking ρ_*X*_ = 0.23 (Figure 2), assuming ρ_B_2__ ≈ 1, and approximating the vertical electronic energy with the adiabatic energy, we estimate ϵ_aa_ ≈ −66 MHz, within a factor of 2 of the experimental value (ϵ_aa_ = −39 MHz). The true vertical electronic energy is strictly larger than the adiabatic value and in fact is probably substantially greater owing to the large difference between the equilibrium geometries of the X̃ and Ã states.^5^ Indeed, we have estimated the Ã−X̃ vertical energy using the EOMIP-CCSD/cc-pVTZ method^34^ to be 2.61 eV. This reduces our rough single-perturber estimate to ϵ_aa_ = −53 MHz.

The “A_1_” excited state in 2-furanyloxy will also have *A*′ electronic symmetry and has not yet been identified experimentally. (It is likely that multiple excited states possess significant O­(2p_*a*_) hole population.) The vertical energy to the first such state in phenoxy is about 5 eV.^10^ If we adopt this value for 2-furanyloxy and account for differences in the *B*/*A* ratio, then we would predict that ϵ_bb_/ϵ_aa_ ≈ 0.24, which under-predicts the experimental ratio of 0.38.

The small absolute magnitudes of the measured ϵ_cc_ and ϵ_ab_ parameters are also consistent with our expectations. ϵ_cc_ is small because the O­(2p_*c*_) π orbital has zero angular momentum projected along the molecular *c* axis and thus matrix elements of *L*_c_ (the electronic orbital angular momentum) are zero to a first approximation. ϵ_ab_ is small because of the approximately orthorhombic local electronic environment near O_2_. Taken together, these crude estimates confirm that the spin-rotation fine structure of 2-furanyloxy is consistent with a small but non-zero residual radical character on the oxy center. [We also note that the ab initio ϵ_*ij*_ predictions (Table 2) are generally quite good, within 1–3 MHz of the experimental values. Interestingly, however, they are systematically too positive compared to the true values. We observed a similar systematic theoretical bias in the spin-rotation structure of phenoxy.^10^ It is unclear to us which aspect of the theoretical treatment leads to this systematic error—partial spin contamination of the UHF reference wavefunction, incomplete basis set effects, terms in the molecular fine structure Hamiltonian neglected in the spin-rotation treatment, zero-point vibrational motion effects, etc.]

We conclude this discussion with a comparison of the structure and unpaired spin distribution of 2-furanyloxy with that of phenoxy, whose microwave spectrum has been characterized with a similar level of analysis.^10^ In both radicals, the electron hole mostly moves from the O atom into the neighboring π system, resulting in the dominant lactone-like form of 2-furanyloxy and the quinone-like form of phenoxy. However, the residual unpaired spin population on the O atom in phenoxy (0.41) is much larger than that in 2-furanyloxy (0.23). That is, phenoxy has decidedly more alkoxy radical character than 2-furanyloxy. Although this may seem counterintuitive given that the six-membered ring in phenoxy has more “room” to accept the electron hole compared to the five-membered ring in 2-furanyloxy, it is consistent with the qualitative resonance structure predictions. 2 of the 5 resonance structures of phenoxy (40%) place the unpaired electron on O, whereas only 1 of the 3 resonance structures of 2-furanyloxy (33%) do so. The difference in alkoxy character is borne out by the much longer C=O bond length in phenoxy (1.247 Å vs 1.203 Å),^10^ the much smaller Ã−X̃ electronic excitation energy (1.06 eV vs 2.14 eV),^5,35^ and the much larger O–CH_3_ bond dissociation energy of the parent methoxy species, i.e., that of anisole (64 kcal/mol)^36^ vs 2-methoxyfuran (45 kcal/mol^3^). Consequently, it is expected that the probability of radical–radical recombination reactions at the O site is considerably higher for phenoxy than for 2-furanyloxy. This comparison extends also to the spectroscopic fine structure of these radicals. That the ϵ_aa_/*A* ratio for phenoxy (−0.018) is a factor of 4 larger than that of 2-furanyloxy (−0.0042) is largely attributable to the increased O spin population and the correspondingly enhanced spin–orbit effects.

## Conclusions

The cm-wave rotational spectrum of 2-furanyloxy radical has been precisely and comprehensively characterized. Our results provide detailed insights into the delocalized unpaired π electron and the resulting description of 2-furanyloxy as an allylic lactone. The accurate spectroscopic parameters enable the rest transition frequencies up to 40 GHz to be calculated to ∼1 kHz accuracy, providing the spectroscopic foundation for quantifying the laboratory and interstellar chemistry of 2-furanyloxy and its role in the oxidation of aromatic rings. This work also motivates a laboratory search for the microwave spectrum of the elusive allyl radical. The characterization of its rotational and hyperfine structure would enable a direct comparison with allylic fragments in 2-furanyloxy and other related π radicals.

## Supplementary Material



## Notes

SPFIT/SPCAT input and output files: 2fur.lin, experimental line list; 2fur.par, SPFIT parameter input file; 2fur.fit, fit results; 2fur.var, SPCAT parameter input file; 2fur.int, SPCAT dipole input file; 2fur.cat, calculated catalog (ZIP)
